# Supplementary material for: HDAC6-Selective Inhibitor Overcomes Bortezomib Resistance in Multiple Myeloma
Source: Int J Mol Sci. 2021 Jan 29;22(3):1341. doi: 10.3390/ijms22031341 (PMC7866276; doi:10.3390/ijms22031341)
Supplement: Supplementary file 1 [file ijms-22-01341-s001.pdf]

Lee et.al. Figure S1

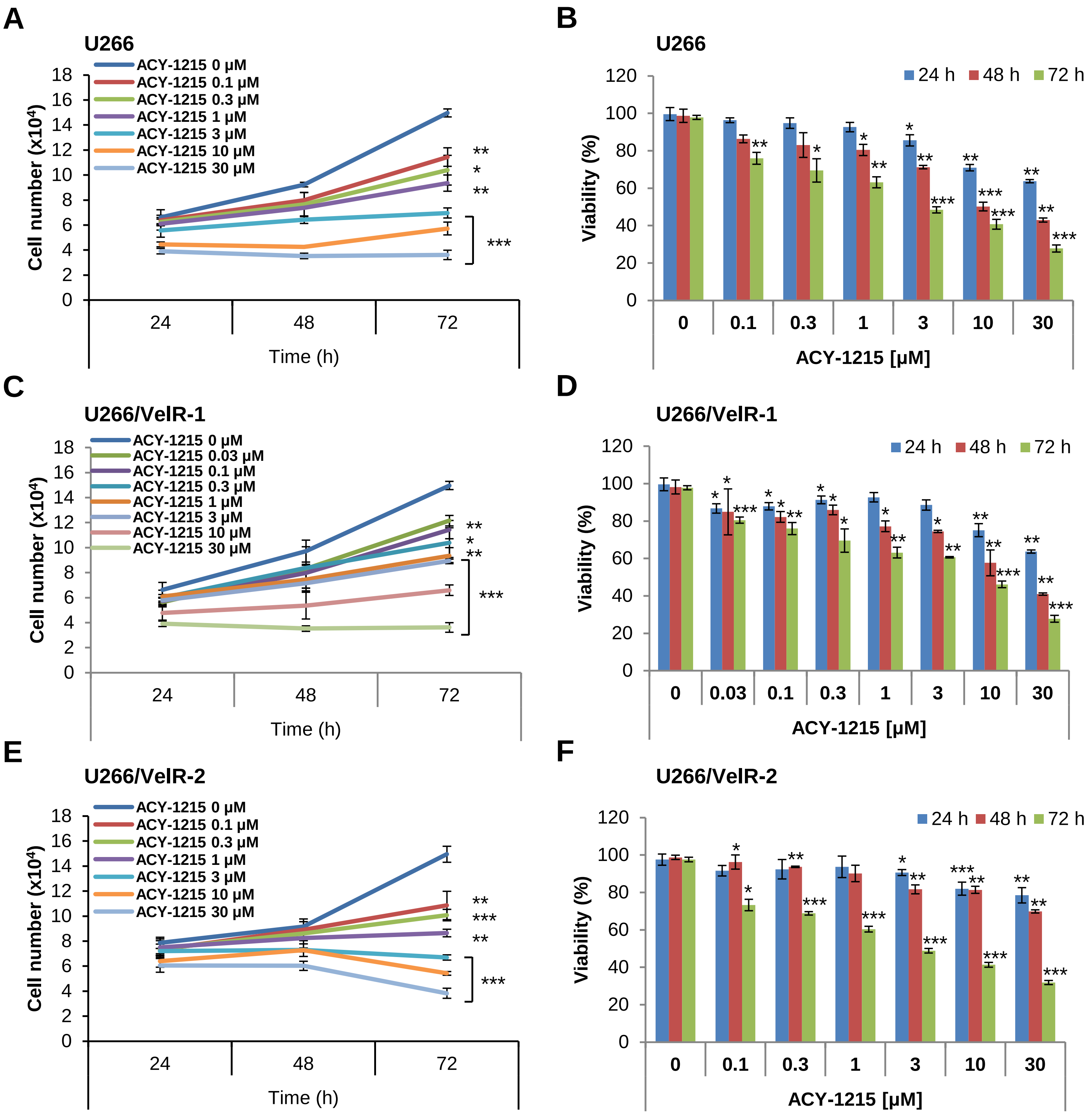

G

72 h	ACY-1215 (μM)	
	GI_{50}	IC_{50}
U266	2.36	2.36
U266/VelR-1	6.91	7.82
U266/VelR-2	2.49	2.58

Figure S1. ACY-1215, an HDAC6-selective inhibitor, suppresses cell growth and viability in BTZ-sensitive and BTZ-resistant U266 MM cells

(**A,B**) BTZ-sensitive U266 and (**C-F**) BTZ-resistant U266 (U266/VelR-1, U266/VelR-2) MM cells were treated with indicated concentrations of ACY-1215 or 0.1% DMSO (control) for 72 h, and CCK-8 assays were performed to analyze cell growth and viability. Cell counts were estimated indirectly from a standard curve generated using solutions of known cell counts. Absorbance was normalized to that of the negative control at each time interval. Data are shown as mean ± SD from three independent experiments (n = 3). Student' *t*-test, * p <0.05, ** p <0.01, and *** p <0.001 vs. the DMSO control. (**G**) The IC_{50} and GI_{50} values for ACY-1215 in BTZ-sensitive and BTZ-resistant U266 cells using GraphPad prism.

Lee et.al. Figure S2

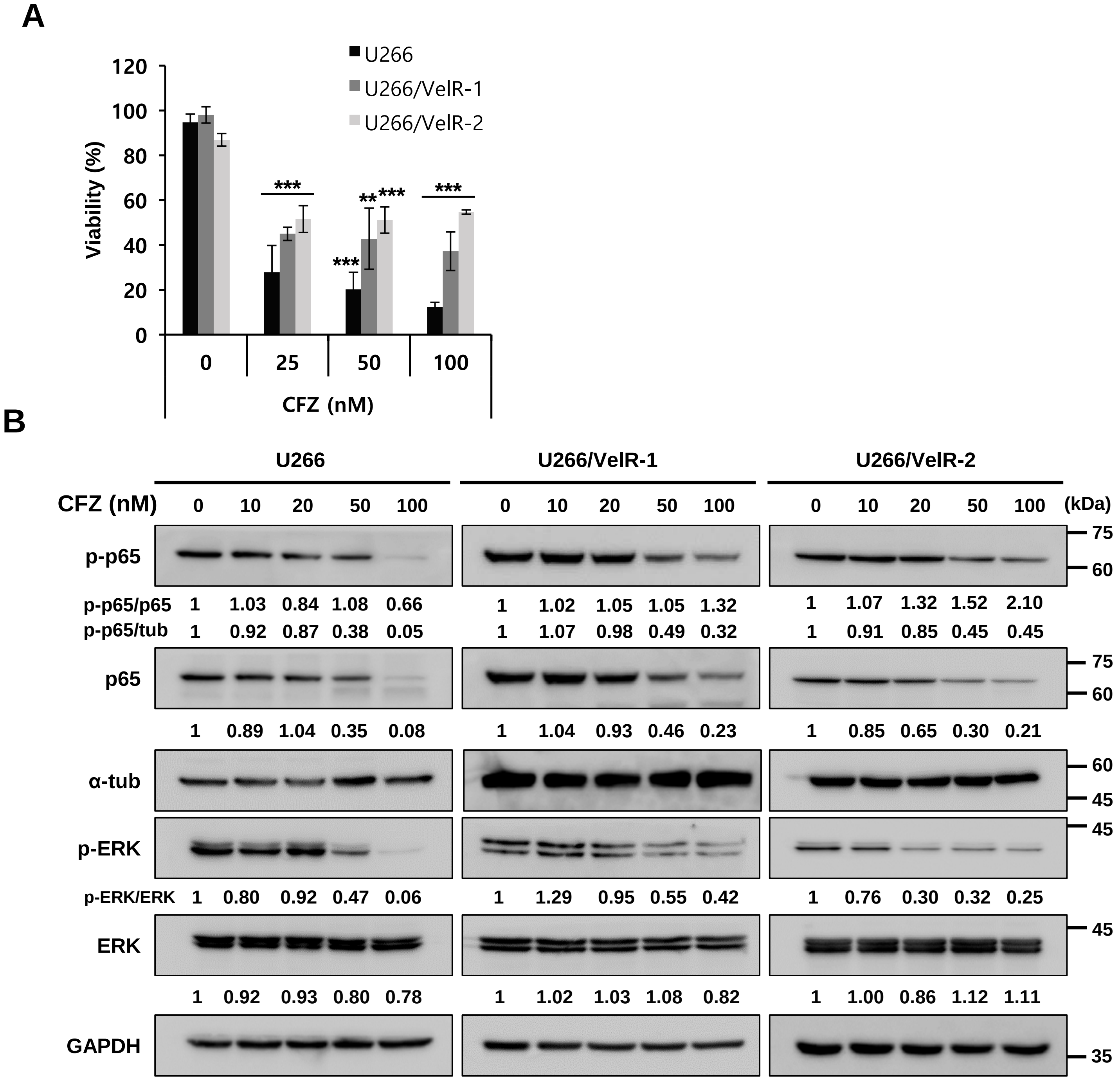

Figure S2. Effects of CFZ on BTZ-resistant U266/VelR MM cells

(**A**) Both BTZ-sensitive U266 and BTZ-resistant U266 (U266/VelR-1 and U266/VelR-2) MM cells were treated with indicated concentrations of CFZ (25, 50, 100 nM) for 72 h, and CCK-8 assays were performed to analyze cell viability. Data represent mean ± SD (n = 3). Student' *t*-test, ** p <0.01 and *** p <0.001 vs. the DMSO control. (**B**) Immunoblotting analysis of both BTZ-sensitive U266 and BTZ-resistant U266 (U266/VelR-1 and U266/VelR-2) MM cells treated with 0.01% DMSO or indicated concentrations of CFZ (10, 20, 50, and 100 nM) for 24 h. Relative protein expression levels were semi-quantified by densitometric analysis of the blots. α-Tub and GAPDH were used as equal loading controls. The abundance of the indicated proteins was semi-quantified relative to α-tub or GAPDH, and control levels were set at 1.

Lee et.al. Figure S3

A

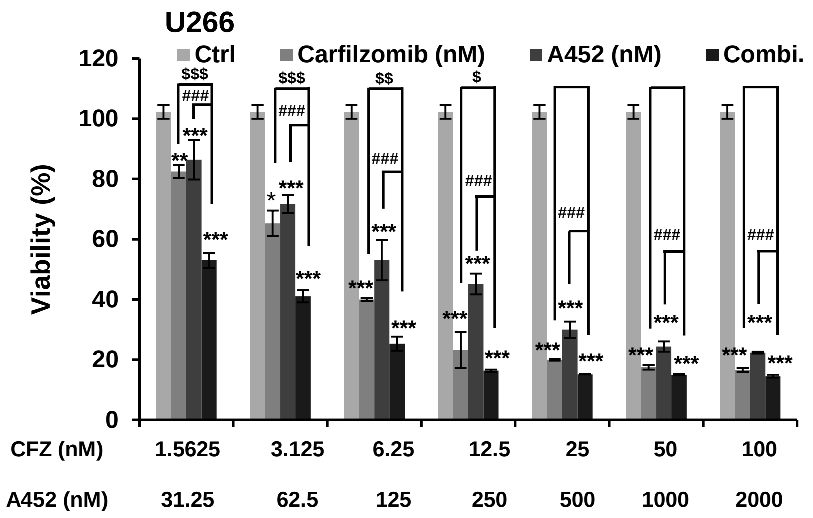

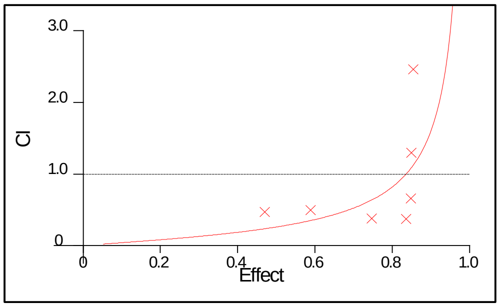

A452 (nM)	CFZ (nM)	Effect	CI
31.25	1.5625	0.469879	0.47
62.5	3.125	0.589369	0.497
125	6.25	0.747357	0.383
250	12.5	0.836564	0.371
500	25	0.848602	0.659
1000	50	0.849852	1.301
2000	100	0.855016	2.465

B

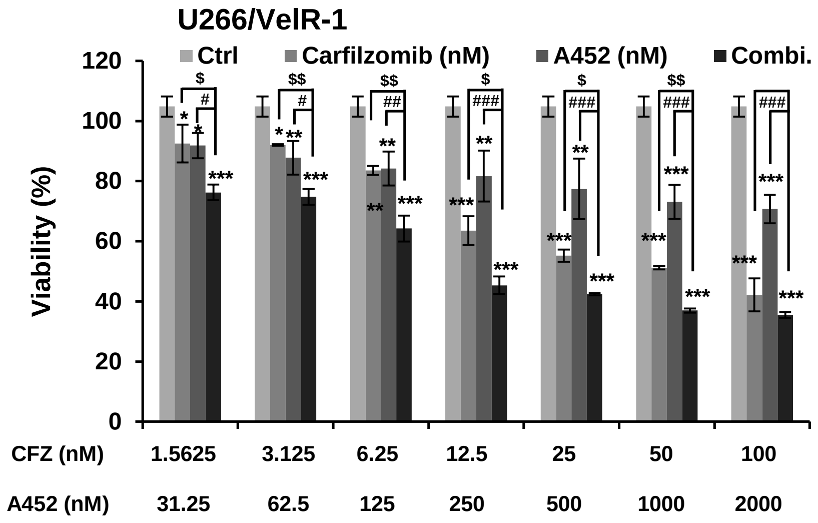

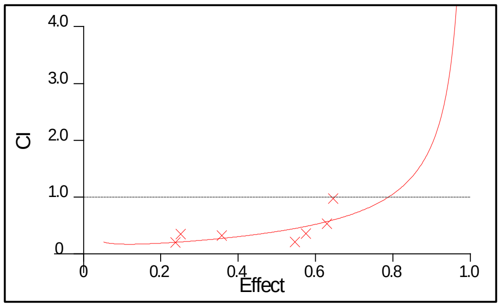

A452 (nM)	CFZ (nM)	Effect	CI
31.25	1.5625	0.237551	0.2
62.5	3.125	0.252132	0.351
125	6.25	0.357614	0.322
250	12.5	0.546424	0.214
500	25	0.575947	0.363
1000	50	0.630293	0.533
2000	100	0.645288	0.976

Figure S3. Cotreatment with A452 and CFZ triggers synergistic cytotoxicity in both BTZ-sensitive and BTZ-resistant U266 MM cells

(**A**) BTZ-sensitive U266 and (**B**) BTZ-resistant U266 (U266/VelR-1) MM cells were treated with 0.1% DMSO (control), A452, and CFZ or in combination with these compounds as indicated for 72 h. Combination treatments were then performed in U266 (**A**) and BTZ-resistant U266 (**B**) MM cells maintaining a constant ratio between the dose of the A452 and CFZ, and cell viability was assessed at 72 h by CCK-8 assay. The combination index (CI) value and the relative fraction affected (F_A) were determined at each dose combination (actual) and a simulation was run to estimate the CI value and confidence interval (---) across the entire F_A range (simulation). CI < 1, CI = 1, and CI >1 indicate synergistic, additive, and antagonistic effects, respectively. CI was calculated by the CalcuSyn software program. Data present as mean ± SD (*n* = 3). * *p* <0.05, ** *p* <0.01, and *** *p* <0.001 *vs*. the DMSO control; $^{\$}$ *p* <0.05, $^{\$\$}$ *p* <0.01, and $^{\$\$\$}$ *p* <0.001 *vs*. CFZ-treated group $^{\#}$ *p* <0.05, $^{\#\#}$ *p* <0.01, and $^{\#\#\#}$ *p* <0.001 *vs*. A452-treated group; two-way analysis of variance (ANOVA) test.

Lee et.al. Figure S4

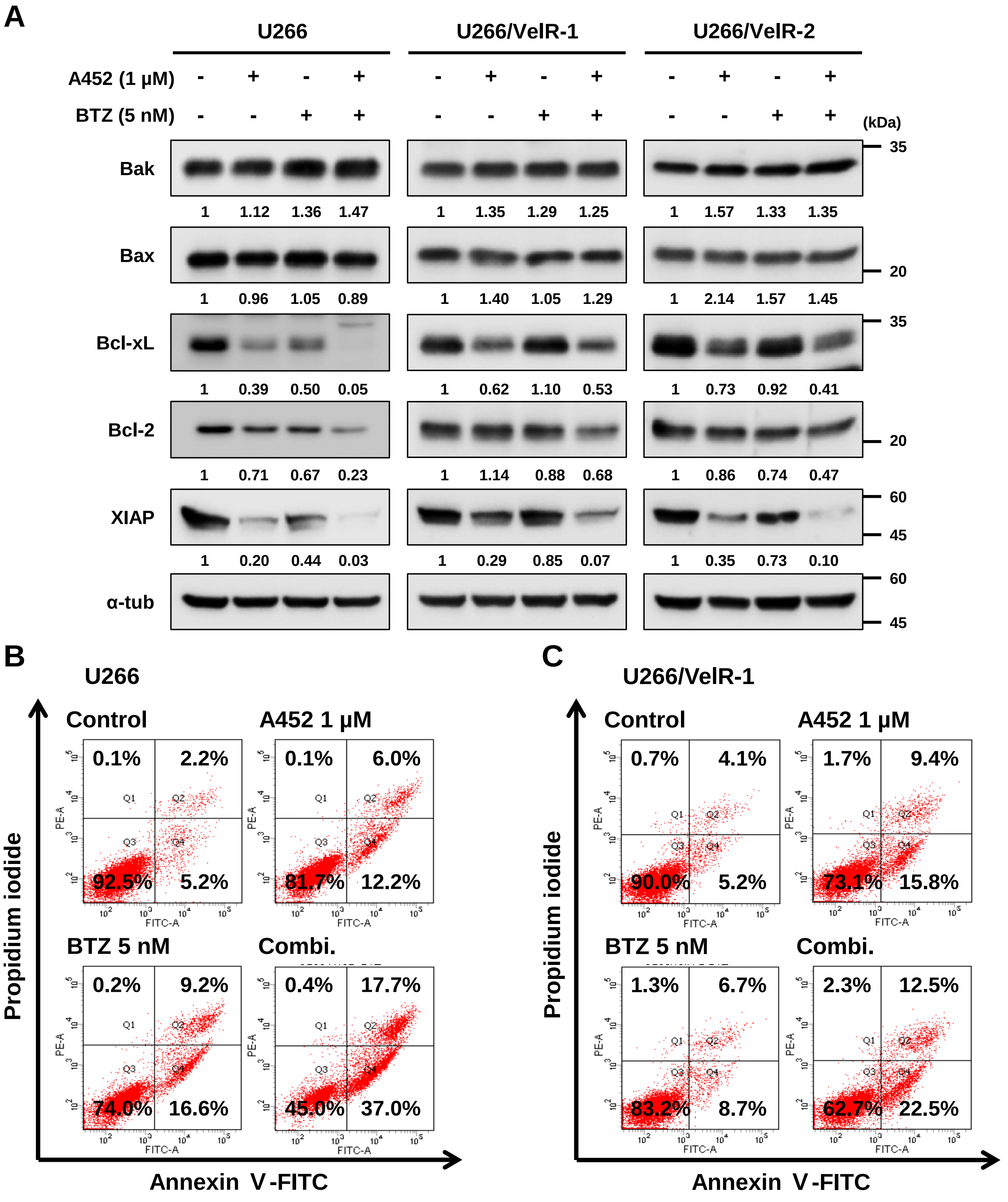

Figure S4. Cotreatment with BTZ and A452 leads to synergistic apoptosis induction

(**A**) BTZ-sensitive U266 and BTZ-resistant U266 (U266/VelR-1) MM cells were treated with 0.1% DMSO (control), A452 (1 μM), and BTZ (5 nM) or in combination with these compounds as indicated for 24 h. Apoptotic markers were identified by immunoblotting using whole-cell lysates. α-Tub was used as an equal loading control. The abundance of the indicated proteins was semi-quantified relative to α-tub; control levels were set at 1. (**B**) BTZ-sensitive U266 and (**C**) BTZ-resistant U266/VelR-1 MM cells were treated with 0.1% DMSO (control), A452 (1 μM), and BTZ (5 nM) or in combination with these compounds as indicated for 24 h. Cell death was assessed by flow cytometry and Annexin V/PI staining (*n* = 3).

Lee et.al. Figure S5

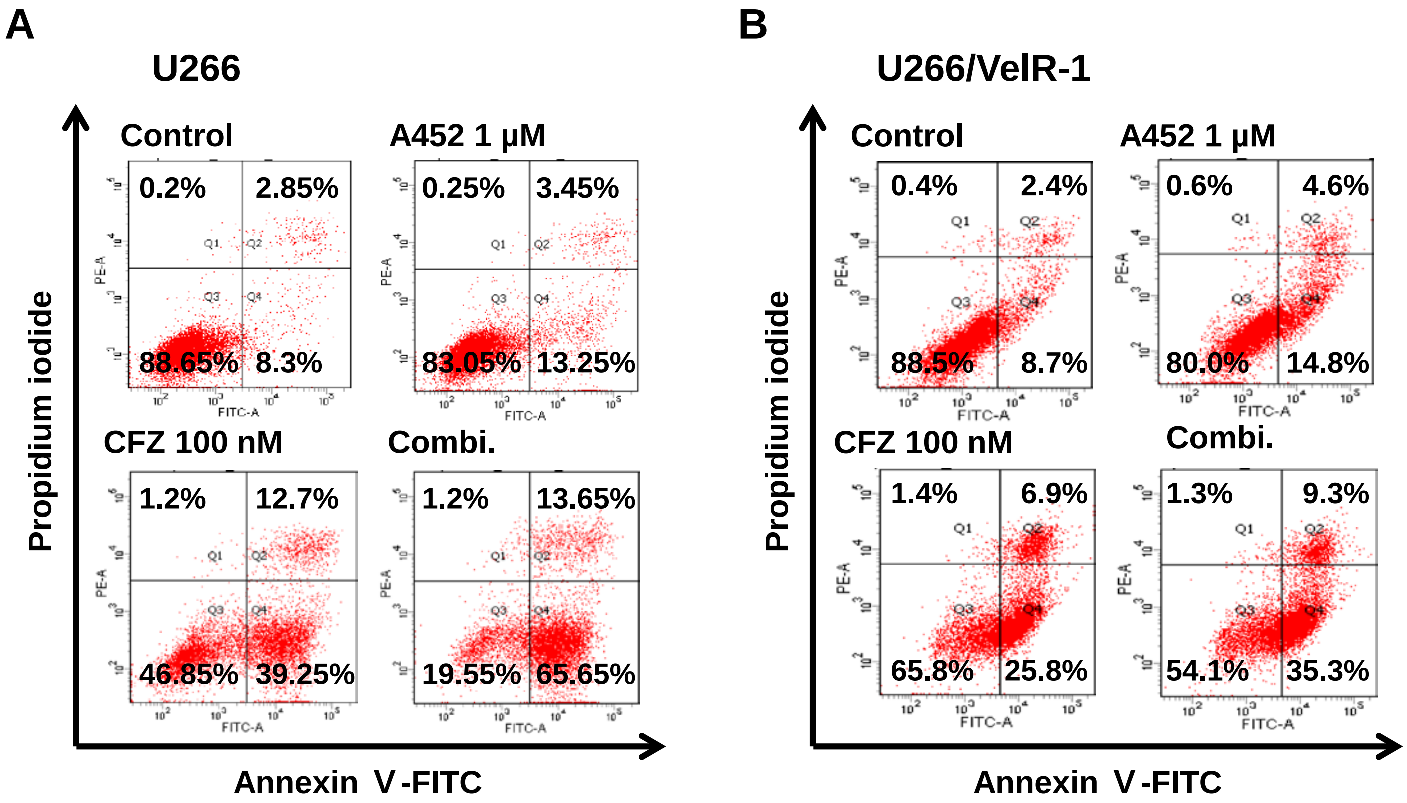

Figure S5. Cotreatment with CFZ and A452 leads to synergistic apoptosis induction

(**A**) BTZ-sensitive U266 and (**B**) BTZ-resistant U266 (U266/VelR-1) MM cells were treated with 0.1% DMSO (control), A452 (1 μM), and CFZ (100 nM) or in combination with these compounds as indicated for 24 h. Cell death was assessed by flow cytometry and Annexin V/PI staining (*n* = 3).
